# Impact of Atrioventricular Valve Intervention at Each Stage of Single Ventricle Palliation

**DOI:** 10.1177/21501351241269924

**Published:** 2024-09-05

**Authors:** John D. Vossler, Aaron W. Eckhauser, Eric R. Griffiths, Reilly D. Hobbs, Linda M. Lambert, Lloyd Y. Tani, Niharika Parsons, Robert H. Habib, Jeffrey P. Jacobs, Marshall L. Jacobs, S. Adil Husain

**Affiliations:** 1 Division of Cardiovascular and Thoracic Surgery, Department of Surgery, 8784 University of California, San Diego, CA, USA; 2 Division of Cardiothoracic Surgery, Department of Surgery, 14434 University of Utah, Salt Lake City, UT, USA; 3 23188 Primary Children's Hospital, Heart Center, Salt Lake City, UT, USA; 4 Division of Pediatric Cardiology, Department of Pediatrics, 8784 University of Utah, Salt Lake City, UT, USA; 5 Research and Analytic Center, 50362 The Society of Thoracic Surgeons, Chicago, IL, USA; 6 Division of Thoracic and Cardiovascular Surgery, Department of Surgery, 3463 University of Florida, Gainesville, FL, USA; 7 Division of Cardiac Surgery, Department of Surgery, 1466 Johns Hopkins University, Baltimore, MD, USA

**Keywords:** CHD, univentricular heart, congenital heart disease, congenital heart surgery, database (all types), functionally univentricular heart, outcomes (includes mortality, morbidity), palliation, statistics, regression analysis, morbidity, mortality

## Abstract

**Background:** Significant atrioventricular valve dysfunction can be associated with mortality or need for transplant in functionally univentricular heart patients undergoing staged palliation. The purposes of this study are to characterize the impact of concomitant atrioventricular valve intervention on outcomes at each stage of single ventricle palliation and to identify risk factors associated with poor outcomes in these patients. **Methods:** The Society of Thoracic Surgeons Congenital Heart Surgery Database was queried for functionally univentricular heart patients undergoing single ventricle palliation from 2013 through 2022. Separate analyses were performed on cohorts corresponding to each stage of palliation (1: initial palliation; 2: superior cavopulmonary anastomosis; 3: Fontan procedure). Bivariate analysis of demographics, diagnoses, comorbidities, preoperative risk factors, operative characteristics, and outcomes with and without concomitant atrioventricular valve intervention was performed. Multiple logistic regression was used to identify predictors associated with operative mortality or major morbidity. **Results:** Concomitant atrioventricular valve intervention was associated with an increased risk of operative mortality or major morbidity for each cohort (cohort 1: 62% vs 46%, *P* < .001; cohort 2: 37% vs 19%, *P* < .001; cohort 3: 22% vs 14%, *P* < .001). Black race in cohort 1 (odds ratio [OR] 3.151, 95% CI 1.181-9.649, *P* = .03) and preterm birth in cohort 2 (OR 1.776, 95% CI 1.049-3.005, *P* = .032) were notable predictors of worse morbidity or mortality. **Conclusions:** Concomitant atrioventricular valve intervention is a risk factor for operative mortality or major morbidity at each stage of single ventricle palliation. Several risk factors are associated with these outcomes and may be useful in guiding decision-making.

## Introduction

Significant atrioventricular valve (AVV) dysfunction occurs in 10% to 25% of functionally univentricular heart (FUVH) patients^1,2^ and is well-known to be an independent risk factor for death and transplant in these patients.^1,3–9^ Successful AVV repair significantly improves their long-term outcomes^6,8,10–15^ and may return them to the same risk profile as FUVH patients without AVV dysfunction.^9,11,16^ However, successful, durable AVV repair is difficult to achieve,^1,6,9,10,13,14^ and AVV repair itself may be a risk factor for death or transplantation.^17,18^ Thus, these patients face a dilemma regarding optimal treatment strategy.

An aggressive approach of early AVV repair in FUVH patients who are otherwise acceptable single ventricle palliation (SVP) candidates is advocated by many,^1,4,5,18^ while others recommend a selective approach with early consideration of heart transplantation.^3,6,19^ It is unclear which strategy is optimal, and it is unknown which factors should be considered for a selective approach. The aims of this study are to characterize the impact on outcomes of concomitant AVV intervention at each stage of SVP and to identify predictors of poor outcomes in patients undergoing concomitant AVV intervention at each stage of SVP.

## Patients and Methods

### Design and Data Source

The Society of Thoracic Surgeons Congenital Heart Surgery Database (STS-CHSD) was used to perform a series of independent retrospective cohort analyses at each stage of SVP. The STS-CHSD contains audit-validated, procedure-level data from over 90% of congenital heart surgery cases performed in the United States.^20^ Analyses were conducted by the STS Research and Analytic Center. This study was determined to be exempt research by the Institutional Review Board (Advarra; Protocol #Mod01760092).

### Patient Cohorts

**Figure 1.**
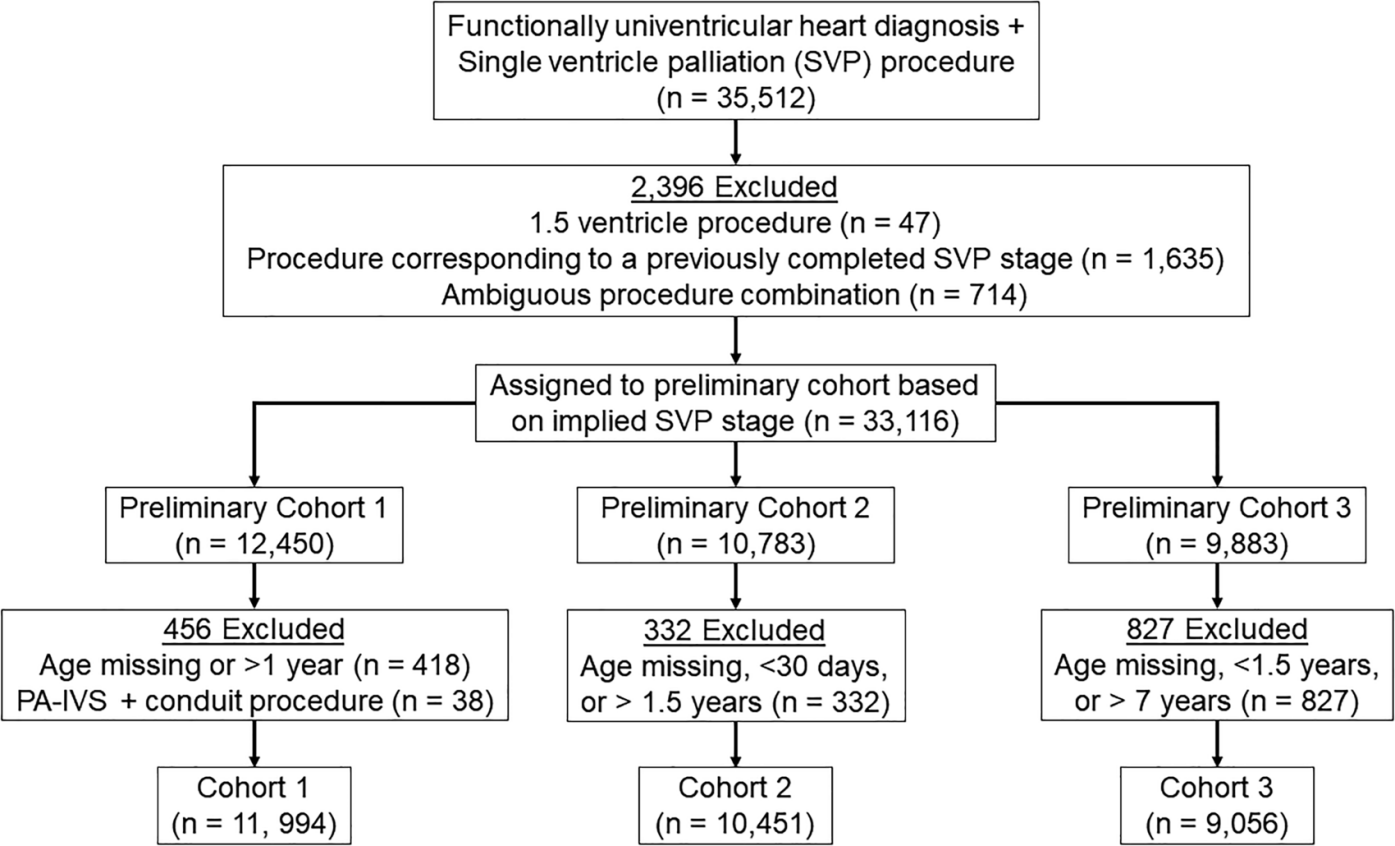
Cohort creation diagram. PA-IVS, pulmonary atresia with intact ventricular septum; SVP, single ventricle palliation; PA-IVS, pulmonary atresia with intact ventricular septum.

The STS-CHSD was queried for patients with a FUVH diagnosis undergoing a procedure consistent with SVP from January 1, 2013, to December 31, 2022 (Supplemental Table 1). Patients undergoing 1.5 ventricle repair or a procedure corresponding to a previously completed SVP stage were excluded. Patients undergoing procedure combinations with ambiguous SVP stage were manually adjudicated to a single stage or excluded (Supplemental Table 2). Patients were then assigned to preliminary cohorts based on SVP stage. Cohort 1 included initial palliation procedures such as Norwood and hybrid stage 1 procedures, cohort 2 included superior cavopulmonary anastomosis procedures, and cohort 3 included Fontan procedures (Supplemental Table 1). Patients undergoing concomitant aortopulmonary amalgamation and superior cavopulmonary anastomosis were included in cohort 2 and assigned an operation value of “Comprehensive Stage 2” (Supplemental Table 2). Patients were excluded from cohort 1 if their age was missing or >1 year. Patients with pulmonary atresia with intact ventricular septum undergoing a conduit procedure were excluded from cohort 1. Patients whose age was missing, <30 days, or >1.5 years were excluded from cohort 2. Patients whose age was missing, <1.5 years, or >7 years were excluded from cohort 3 (Figure 1). These cohorts are independent of one another and unrelated except that they correspond to patients along with the same clinical pathway. Thus, each cohort was analyzed independently with no attempt made to compare across cohorts or to perform longitudinal analysis.

### Outcomes

A composite end point of operative mortality and/or major morbidity was the primary outcome analyzed. Operative mortality was defined as death within 30 days of procedure or prior to hospital discharge. Major morbidity was defined as the occurrence of any of the following within 30 days of procedure: renal failure requiring dialysis, neurologic deficit persisting at discharge, arrhythmia requiring permanent pacemaker, mechanical circulatory support, phrenic nerve injury, or unplanned reoperation.^21^ The individual components of the primary outcome were analyzed as secondary outcomes.

### Analysis

Within each cohort, patients were stratified by the occurrence of concomitant AVV intervention (Supplemental Table 3). Bivariate analysis of demographics, diagnosis, comorbidities, preoperative risk factors, operative characteristics, and outcomes with and without concomitant AVV intervention was conducted within each cohort using an appropriate test of statistical inference for each variable (χ^2^, Fisher exact, Wilcoxon rank sum). Multivariable analysis of the concomitant AVV intervention subgroup of each cohort was conducted to find preoperative predictors of composite operative mortality or major morbidity by multiple logistic regression. Models were created by backward stepwise regression starting with a fully saturated model. Age group, weight, ventricular dominance, and operation were included in the minimum model. Statistical significance was assigned to *P*-values <.05.

## Results

### Cohort 1

Complete bivariate analysis of demographics, diagnoses, comorbidities, preoperative risk factors, and operative characteristics of those with and without concomitant AVV intervention for cohort 1 is reported in Supplemental Table 6. Concomitant AVV intervention was more prevalent among cohort 1 patients who were in age group 1 to 12 months (3.7% vs 2.0% [age group 0-1 month], *P* < .001), right ventricle (RV) (2.8%) or ambiguous ventricle (2.4%) dominant (vs 1.0%; left ventricle [LV] dominant, *P* < .001), and undergoing Norwood procedure (2.8% vs 1.3% [Shunt] vs 1.9% [pulmonary artery band] vs 0.6% [other], *P* < .001). Cohort 1 patients undergoing concomitant AVV intervention were heavier (3.33 kg vs 3.15 kg, *P* < .001) and had a higher prevalence of prior AVV intervention (1.1% vs 0.1%, *P* = .009), preoperative mechanical ventilation (50% vs 36%, *P* < .001), and reoperative surgery (18% vs 7.8%, *P* < .001). Total operative time (355 min vs 263 min, *P* < .001), cardiopulmonary bypass time (182 vs 121 min, *P* < .001), and cross clamp time (78 vs 42 min, *P* < .001) were longer for patients undergoing concomitant AVV intervention.

Bivariate analysis of outcomes with and without concomitant AVV intervention for cohort 1 is reported in Table 1. Concomitant AVV intervention was associated with a higher occurrence of composite operative mortality or major morbidity (62% vs 46%, *P* < .001) as well as all components of the composite outcome except neurologic deficit (3.0% vs 2.0%, *P* = .2) and paralyzed diaphragm (4.1% vs 3.1%, *P* = .4).

**Table 1.** Cohort 1: Bivariate Analysis of Outcomes Versus Concomitant AVV Intervention.

	Overall, n (%)	Concomitant AVV Intervention		*P* value
		No, n (%)	Yes, n (%)	
Number of patients, n	11,994	11,726	268	
Composite operative mortality or major morbidity	5,554 (46%)	5,389 (46%)	165 (62%)	<.001
Operative mortality	1,576 (13%)	1,499 (13%)	77 (29%)	<.001
Unknown	56	54	2	
Mechanical circulatory support	1,707 (14%)	1,640 (14%)	67 (25%)	<.001
Unplanned reoperation	4,588 (38%)	4,463 (38%)	125 (47%)	.004
Permanent pacemaker	136 (1.1%)	126 (1.1%)	10 (3.7%)	<.001
Renal failure	597 (5.0%)	576 (4.9%)	21 (7.8%)	.03
Neurologic deficit	238 (2.0%)	230 (2.0%)	8 (3.0%)	.2
Paralyzed diaphragm	379 (3.2%)	368 (3.1%)	11 (4.1%)	.4

Abbreviation: AVV, atrioventricular valve.

Multivariable analysis of predictors of composite operative mortality or major morbidity for cohort 1 patients undergoing concomitant AVV intervention is reported in Table 2. Predictors independently associated with a higher occurrence of composite mortality or major morbidity are black race (reference white, odds ratio [OR] = 3.151, 95% confidence interval [CI] = 1.181-9.649, *P* = .03) and ambiguous ventricular dominance (reference LV, OR = 3.355, 95% CI = 1.242-9.408, *P* = .018). Each marginal increase of 1 kg of weight is independently associated with a decreased occurrence of the composite outcome (OR = 0.627, 95% CI = 0.457-0.843, *P* = .003).

**Table 2.** Cohort 1: Multivariable Analysis of Predictors of Composite Operative Mortality or Major Morbidity for Patients Undergoing Concurrent AVV Intervention.

Predictor	Odds ratio	95% confidence interval	*P* value
Age group			
0-1 month	1.0 (reference)	—	—
1-12 months	0.846	0.409-1.776	.654
Race			
White	1.0 (reference)	—	—
Asian	1.258	0.281-6.103	.764
Black	3.151	1.181-9.649	.03
Other/Multiple	1.646	0.887-3.123	.119
Weight (kg)	0.627	0.457-0.843	.003
Ventricular dominance			
Left ventricle	1.0 (reference)	—	—
Right ventricle	1.293	0.473-3.534	.614
Ambiguous	3.355	1.242-9.408	.018
Operation			
Norwood	1.0 (reference)	—	—
Shunt	1.042	0.392-2.847	.934
PA Band	0.574	0.244-1.343	.199
Other	734,332	0.000-NA	.988

Abbreviation: PA, pulmonary artery.

### Cohort 2

Complete bivariate analysis of demographics, diagnoses, comorbidities, preoperative risk factors, and operative characteristics of those with and without concomitant AVV intervention for cohort 2 is reported in Supplemental Table 7. Concomitant AVV intervention was more prevalent among cohort 2 patients who were RV (8.1%) or ambiguous ventricle (8.1%) dominant (vs 3.1% [LV dominant], *P* < .001) and undergoing urgent surgery (9.4% vs 5.9% [elective] vs 7.0% [salvage/emergent], *P* < .001). Cohort 2 patients undergoing concomitant AVV intervention were younger (157 vs 162 days, *P* = .014), lighter (6.06 vs 6.28 kg, *P* = .001), and had a higher prevalence of prior AVV intervention (7.8% vs 1.2%, *P* < .001), preoperative mechanical ventilation (20% vs 10%, *P* < .001), and other preoperative risk factors (Supplemental Table 5) (54% vs 46%, *P* < .001). Total operative time (317 vs 239 min, *P* < .001), cardiopulmonary bypass time (143 vs 95 min, *P* < .001), and cross clamp time (45 vs 0 min, *P* < .001) were longer for patients undergoing concomitant AVV intervention.

Bivariate analysis of outcomes with and without concomitant AVV intervention for cohort 2 is reported in Table 3. Concomitant AVV intervention was associated with a higher occurrence of composite operative mortality or major morbidity (37% vs 19%, *P* < .001) as well as all components of the composite outcome except neurologic deficit (1.0% vs 0.8%, *P* = .4) and paralyzed diaphragm (4.2% vs 3.1%, *P* = .12).

**Table 3.** Cohort 2: Bivariate Analysis of Outcomes Versus Concomitant AVV Intervention.

	Overall, n (%)	Concomitant AVV Intervention		*P* value
		No, n (%)	Yes, n (%)	
Number of patients, n	10,451	9,784	667	
Composite operative mortality or major morbidity	2,081 (20%)	1,834 (19%)	247 (37%)	<.001
Operative mortality	341 (3.3%)	262 (2.7%)	79 (12%)	<.001
Unknown	12	10	2	
Mechanical circulatory support	223 (2.1%)	173 (1.8%)	50 (7.5%)	<.001
Unplanned reoperation	1,750 (17%)	1,557 (16%)	193 (29%)	<.001
Permanent pacemaker	59 (0.6%)	43 (0.4%)	16 (2.4%)	<.001
Renal failure	58 (0.6%)	45 (0.5%)	13 (1.9%)	<.001
Neurologic deficit	81 (0.8%)	74 (0.8%)	7 (1.0%)	.4
Paralyzed diaphragm	333 (3.2%)	305 (3.1%)	28 (4.2%)	.12

Abbreviation: AVV, atrioventricular valve.

Multivariable analysis of predictors of composite operative mortality or major morbidity for cohort 2 patients undergoing concomitant AVV intervention is reported in Table 4. Predictors independently associated with a higher occurrence of composite mortality or major morbidity are preterm birth (OR = 1.776, 95% CI = 1.049-3.005, *P* = .032), presence of other preoperative risk factors (Supplemental Table 5) (OR = 1.445, 95% CI = 1.033-2.028, *P* = .032), mechanical ventilation prior to surgery (OR = 2.463, 95% CI = 1.613-3.786, *P* < .001), and comprehensive stage 2 procedure (reference superior cavopulmonary anastomosis, OR = 2.476, 95% CI = 1.143-5.494, *P* = .022).

**Table 4.** Cohort 2: Multivariable Analysis of Predictors of Composite Operative Mortality or Major Morbidity for Patients Undergoing Concurrent AVV Intervention.

Predictor	Odds ratio	95% confidence interval	*P* value
Age group			
1-4 months	1.354	0.874, 2.106	.175
4-8 months	1.0 (reference)	—	—
8-18 months	0.606	0.346, 1.027	.07
Hispanic	0.672	0.442, 1.007	.058
Preterm	1.776	1.049, 3.005	.032
Weight (kg)	1.079	0.967, 1.261	.274
Ventricular dominance			
Left ventricle	1.0 (reference)	—	—
Right ventricle	0.84	0.517, 1.378	.486
Ambiguous	1.172	0.684, 2.022	.564
Chromosomal abnormality	1.514	0.975, 2.345	.063
Other preoperative risk factors^a^	1.445	1.033, 2.028	.032
Mechanical ventilation prior to surgery	2.463	1.613, 3.786	<.001
Operation			
Superior cavopulmonary anastomosis	1.0 (reference)	—	—
Comprehensive stage 2	2.476	1.143, 5.494	.022
Reoperative surgery	1.73	0.933, 3.334	.09

^a^ See Supplemental Table 5.

### Cohort 3

Complete bivariate analysis of demographics, diagnoses, comorbidities, preoperative risk factors, and operative characteristics of those with and without concomitant AVV intervention for cohort 3 is reported in Supplemental Table 8. Concomitant AVV intervention was more prevalent among cohort 3 patients who were RV (10.4%) or ambiguous ventricle (10.8%) dominant (vs 2.8% [LV dominant], *P* < .001). Cohort 3 patients undergoing concomitant AVV intervention were lighter (14.2 vs 14.6 kg, *P* = .005) and had a higher prevalence of prior AVV intervention (7.8% vs 1.2%, *P* < .001), major noncardiac abnormality (22% vs 15%, *P* < .001), and shock at time of surgery (0.4% vs <0.1%, *P* = .004). Total operative time (335 vs 258 min, *P* < .001), cardiopulmonary bypass time (136 vs 89 min, *P* < .001), and cross clamp time (58 vs 0 min, *P* < .001) were longer for patients undergoing concomitant AVV intervention.

Bivariate analysis of outcomes with and without concomitant AVV intervention for cohort 3 is reported in Table 5. Concomitant AVV intervention was associated with a higher occurrence of composite operative mortality or major morbidity (22% vs 14%, *P* < .001) as well as all components of the composite outcome except neurologic deficit (1.8% vs 1.3%, *P* = .3) and paralyzed diaphragm (1.9% vs 1.7%, *P* = .7). Notably, concomitant AVV intervention was associated with a much higher occurrence of permanent pacemaker placement (5.2% vs 1.2%, *P* < .001).

**Table 5.** Cohort 3: Bivariate Analysis of Outcomes Versus Concomitant AVV Intervention.

	Overall, n (%)	Concomitant AVV Intervention		*P* value
		No, n (%)	Yes, n (%)	
Number of patients, n	9,056	8,381	675	
Composite operative mortality or major morbidity	1,326 (15%)	1,176 (14%)	150 (22%)	<.001
Operative mortality	101 (1.1%)	86 (1.0%)	15 (2.2%)	.004
Unknown	8	6	2	
Mechanical circulatory support	120 (1.3%)	100 (1.2%)	20 (3.0%)	<.001
Unplanned reoperation	1,089 (12%)	970 (12%)	119 (18%)	<.001
Permanent pacemaker	136 (1.5%)	101 (1.2%)	35 (5.2%)	<.001
Renal failure	83 (0.9%)	71 (0.8%)	12 (1.8%)	.015
Neurologic deficit	121 (1.3%)	109 (1.3%)	12 (1.8%)	.3
Paralyzed diaphragm	155 (1.7%)	142 (1.7%)	13 (1.9%)	.7

Abbreviation: AVV, atrioventricular valve.

Multivariable analysis of predictors of composite operative mortality or major morbidity for cohort 3 patients undergoing concomitant AVV intervention is reported in Table 6. Predictors independently associated with a higher occurrence of composite mortality or major morbidity are ambiguous ventricular dominance (reference LV, OR = 1.939, 95% CI = 1.036-3.806, *P* = .045) and presence of other preoperative risk factor (Supplemental Table 5) (OR = 1.608, 95% CI = 1.110-2.335, *P* = .012).

**Table 6.** Cohort 3: Multivariable Analysis of Predictors of Composite Operative Mortality or Major Morbidity for Patients Undergoing Concurrent AVV Intervention.

Predictor	Odds ratio	95% confidence interval	*P* value
Age group			
1.5-3 years	1.078	0.676-1.688	.748
3-4.5 years	1.0 (reference)	—	—
4.5-7 years	1.231	0.758-2.006	.4
Weight (kg)	0.974	0.897-1.010	.409
Ventricular dominance			
Left ventricle	1.0 (reference)	—	—
Right ventricle	1.394	0.776-2.647	.286
Ambiguous	1.939	1.036-3.806	.045
Other preoperative risk factor^a^	1.608	1.110-2.335	.012
Mechanical ventilation prior to surgery	3.857	0.880-16.94	.064

^a^ See Supplemental Table 5.

## Comment

Concomitant AVV intervention is associated with an increased risk of operative mortality and major morbidity, including requirement of mechanical circulatory support, unplanned reoperation, permanent pacemaker, and renal failure at all stages of SVP. This is consistent with prior studies showing that AVV dysfunction and concomitant AVV intervention are risk factors for morbidity and mortality in these patients.^1,3–9,17,18^ This study adds to the body of knowledge on the subject by confirming that these findings hold true for short-term outcomes across all FUVH patients undergoing SVP.

Increased weight was found to be associated with a decreased occurrence of composite operative mortality or major morbidity in cohort 1 but not cohorts 2 and 3. This is consistent with the notion that weight is a more important factor when operating on neonates than older infants and children. The finding that a comprehensive stage 2 procedure was independently associated with an increased occurrence of composite operative mortality or major morbidity was expected. A comprehensive stage 2 operation is longer and more technically challenging than a superior cavopulmonary anastomosis and is reserved by some centers for high-risk patients. Therefore, comprehensive stage 2 would be expected to have worse outcomes when compared directly with superior cavopulmonary anastomosis. This study did not examine longitudinal outcomes, so it is impossible to compare the entire pathways leading up to and including these procedures. Thus, we cannot make any conclusion comparing these approaches with SVP. The presence of one or more preoperative noncardiac risk factors (Supplemental Table 5) was identified as independently associated with composite operative mortality or major morbidity in cohorts 2 and 3. It is, however, not possible to objectively speculate as to why any among this diverse list of noncardiac preoperative factors was associated with composite operative mortality or major morbidity.

Black race was identified as being independently associated with composite operative mortality or major morbidity for cohort 1. This is consistent with prior studies showing black race to be a risk factor for poor surgical outcomes.^22^ This finding highlights the need to ensure our databases are well-equipped to study healthcare disparities. Ongoing investigation into the reasons for these differences is critical to improve outcomes.

Preterm birth was identified as being independently associated with composite operative mortality or major morbidity for cohort 2. Preterm patients are known to have parenchymal lung disease that may predispose them to having increased pulmonary vascular resistance (PVR) throughout life. Cohort 1 patients have shunted physiology in the postoperative period, so elevated PVR is not expected to be a large concern for them and may even be helpful depending on their hemodynamic balance. Cohort 2 patients rely on passive blood flow through the pulmonary vasculature and would be expected to be more sensitive to elevated PVR. It has been shown that elevated PA pressure prior to stage 2 is a risk factor for Fontan completion failure,^5^ which is consistent with this finding. Patients included in cohort 3 have already demonstrated that their PVR is low enough for passive pulmonary blood flow, and thus, preterm birth would no longer be expected to be a risk factor for them. Similarly, preoperative mechanical ventilation was found to be independently associated with composite operative mortality or major morbidity for cohort 2. This is consistent with the concept that the condition of the lungs and pulmonary vasculature is a strong driver of stage 2 outcomes. Careful consideration should be given to patients who are preterm or require mechanical ventilation prior to undergoing concomitant AVV intervention during stage 2 SVP.

Ambiguous ventricular dominance was found to be independently associated with composite operative mortality or major morbidity in cohorts 1 and 3. Ambiguous ventricular dominance included patients with unbalanced atrioventricular canal, heterotaxy syndrome, or other as their FUVH diagnosis. All three of these presumably place patients at risk for reasons other than their dominant ventricle morphology and have previously been shown to be risk factors for poor outcomes.^1,19^ Interestingly, RV dominance was not found to be associated with composite operative mortality or major morbidity by multivariable analysis. This is contrary to several prior studies.^1,10,23–26^ However, all of these studies looked at long-term outcomes, whereas this study looked at short-term outcomes. While RV dominance may be an important risk factor for poor long-term outcomes, it does not appear to influence short-term outcomes. Careful consideration should be given to patients with unbalanced atrioventricular canal, heterotaxy syndrome, or other FUVH diagnoses prior to concomitant AVV intervention during stages 1 and 3 SVP.

This study has several limitations. First, its retrospective design precludes conclusions of causality. Next, SVP is a treatment strategy, not a diagnosis or procedure. Thus, it is not directly captured in the STS-CHSD. Single ventricle palliation strategy and stage were inferred from combinations of diagnosis, procedure, and age. It is possible that some patients were improperly included/excluded from the study, particularly the patients excluded for ambiguous SVP stage. Next, the STS-CHSD lacks the data necessary for longitudinal analysis; thus, we were unable to analyze this patient population through the entire SVP pathway. This limitation included the inability to link, with confidence, sequential hospital encounters that may correspond to a given patient, especially across multiple participant institutions. Next, these cohorts are very heterogeneous, and extrapolating these findings to any given patient is challenging. Next, we limited our time frame to the most recent ten years with complete data with the intent of limiting “era” effects. This may have been too broad of a time frame to completely eliminate this effect. Finally, as is the case with all retrospective database analyses, there are several relevant data points that are not captured, not detailed, or incomplete. These include, but are not limited to, ventricular dominance, ventricular function, pre- and postoperative AVV function, hemodynamics, rhythm, AVV repair technique, and long-term outcomes. Moderate to severe AVV regurgitation is captured by the STS-CHSD, but it was missing or unknown for 46% of patients in this study. We therefore reported the variable in our bivariate analysis for completeness but chose to omit it from further analysis.

Concomitant AVV intervention is a risk factor for operative mortality or major morbidity in FUVH patients at each stage of SVP. Several risk factors were identified as being independently associated with these poor outcomes and may be useful in guiding decision-making for these patients. Further investigation is necessary to define the optimal treatment strategy for this challenging patient population.

## Supplemental Material

sj-xlsx-1-pch-10.1177_21501351241269924 - Supplemental material for Impact of Atrioventricular Valve Intervention at Each Stage of Single Ventricle Palliation Supplemental material, sj-xlsx-1-pch-10.1177_21501351241269924 for Impact of Atrioventricular Valve Intervention at Each Stage of Single Ventricle Palliation by John D. Vossler, Aaron W. Eckhauser, Eric R. Griffiths, Reilly D. Hobbs, Linda M. Lambert, Lloyd Y. Tani, Niharika Parsons, Robert H. Habib, Jeffrey P. Jacobs, Marshall L. Jacobs and S. Adil Husain in World Journal for Pediatric and Congenital Heart Surgery

## Abbreviations

AVV: atrioventricular valve
CHSD: congenital heart surgery database
CI: confidence interval
FUVH: functionally univentricular heart
LV: left ventricle
OR: odds ratio
PVR: pulmonary vascular resistance
RV: right ventricle
STS: Society of Thoracic Surgeons
SVP: single ventricle palliation
